# Prevalence and Risk Factors of HIV Drug Resistance in Zimbabwe: Evidence from Zimbabwe Population-Based HIV Impact Assessment (ZIMPHIA) 2020 Survey

**DOI:** 10.3390/tropicalmed9110257

**Published:** 2024-10-28

**Authors:** Munyaradzi Mapingure, Solomon Mukwenha, Innocent Chingombe, Rutendo Birri Makota, Elliot Mbunge, Enos Moyo, Garikayi Chemhaka, John Batani, Brian Moyo, Godfrey Musuka

**Affiliations:** 1 Department of Global Public Health and Family Medicine, Faculty of Medicine and Health Sciences, University of Zimbabwe, Harare 263, Zimbabwe; 2 Texila America University, Georgetown 592, Guyana; smukwenha@yahoo.co.uk; 3 Innovative Public Health and Development, Harare 263, Zimbabwe; ichingombe@yahoo.com; 4 Department of Biomedical Informatics and Biomedical Engineering, Faculty of Medicine and Health Sciences, University of Zimbabwe, Harare 263, Zimbabwe; rutendobbirri@gmail.com; 5 Division of Research, Innovation and Engagement, Mangosuthu University of Technology, Umlazi, Durban 4031, South Africa; mbungeelliot@gmail.com; 6 School of Nursing and Public Health, Faculty of Medicine and Health Sciences, University of Kwazulu Natal, Durban 4031, South Africa; moyoenos@yahoo.co.uk; 7 Department of Statistics and Demography, Faculty of Social Sciences, University of Eswatini, Private Bag 4, Kwaluseni Campus, Kwaluseni 268, Eswatini; bgchem@gmail.com; 8 Faculty of Engineering and Technology, Botho University, Maseru 100, Lesotho; jonj188@gmail.com; 9 AIDS and TB Programmes, Ministry of Health and Child Care, Harare 263, Zimbabwe; moyobk1@gmail.com; 10 International Initiative for Impact Evaluation, Harare 263, Zimbabwe; gmusuka@3ieimpact.org

**Keywords:** HIV, drug resistance, predictors, Zimbabwe, antiretroviral therapy

## Abstract

(1) Background: HIV drug resistance (HIVDR) poses a significant challenge to the effectiveness of antiretroviral therapy and the overall management of HIVand AIDS. Understanding the predictors of HIVDR is critical for developing strategies to mitigate its impact. The objectives of this study were to identify the predictors of HIVDR among Zimbabwe Population-Based HIV Impact Assessment (ZIMPHIA 2020) study participants, a national population-based survey. (2) Methods: Data from people living with HIV who participated in the ZIMPHIA 2020 were used to determine the predictors of HIVDR. (3) Results: The prevalence of HIVDR was 44.9%. Acquired HIVDR was present in 76.1% of people with a virological failure and transmitted resistance is 22.6% in naïve individuals. Factors associated with HIVDR in adjusted analysis were the number of lifetime sexual partners (aOR = 1.03, 95% CI: 1.01–1.06, *p* = 0.017), each additional year since the first HIV positive result (aOR = 1.17, 95% CI: 1.09–1.25, *p* < 0.01), each additional year on ART (aOR = 1.14, 95% CI: 1.06–1.23, *p* = 0.001), initiating ART before 2014 (aOR = 3.08, 95% CI: 1.72–5.49, *p* = 0.020), ever had switched antiretrovirals (aOR = 2.47, 95% CI: 1.15–5.29, *p* = 0.020) or had ever had a viral load test (aOR = 2.54, 95% CI: 1.54–4.17, *p* < 0.001) and a CD4 count < 350 (aOR = 2.04, 95% CI: 1.48–2.83, *p* < 0.01), while age ≥ 50 (aOR = 0.56, 95% CI: 0.32–0.98, 32 *p* = 0.04), condom use at last encounter (OR: 0.49, 95%CI: 0.33–0.73, *p* < 0.001), and not being on ART (aOR = 0.09, 95% CI: 0.06–0.13, *p* < 0.01) were associated with reduced odds of HIVDR. Conclusions: HIVDR was high among the participants. There is a need to address HIVDR and enhance the mechanisms already in place. This study introduces more information that would help in developing targeted interventions to prevent HIVDR and improve patient outcomes.

## 1. Introduction

HIV drug resistance (HIVDR) is a phenomenon where the human immunodeficiency virus (HIV) mutates and becomes resistant to antiretroviral drugs. HIVDR is caused by changes in the genetic structure of HIV that affect the ability of medicines to block the replication of the virus [1,2]. Mutations in the HIV genome can confer resistance to various drug classes, compromising the efficacy of treatment and increasing the risk of disease progression [2].

The emergence of HIVDR can significantly impact the effectiveness of ART programs, leading to increased morbidity, mortality, and transmission. The World Health Organization (WHO) recommends that countries routinely implement nationally representative HIVDR surveys. In 2020, Zimbabwe carried out a national survey called Zimbabwe Population-Based HIV Impact Assessment 2020 (ZIMPHIA 2020) to estimate the HIV incidence and viral load suppression. The prevalence of HIV among adults in Zimbabwe was 12.9%, which corresponds to approximately 1,225,000 adults living with HIV according to the ZIMPHIA 2020 report [3]. It was also found that 86.8% of adults living with HIV were aware of their status, and of those aware, 97.0% were on (ART).

The following Nucleoside/tide Reverse Transcriptase Inhibitors (NRTI) antiretrovirals (ARVs) are available for prescribing in Zimbabwe; Tenofovir Disoproxil Fumarate (TDF)/Tenofovir alafenamide (TAF), Zidovudine (AZT, ZDV), Lamivudine (3TC), Emtricitabine (FTC), Abacavir (ABC). The country’s national guidelines recommend triple combination therapy as the preferred first-line regimen, often including TDF + 3TC + dolutegravir (DTG), and one of the alternative first-line regimens is TDF (TAF) + 3TC (FTC) + efavirenz (EFV) [4]. Viral load testing services are offered free of charge at public health facilities in Zimbabwe. These services were decentralized to provinces in 2016 with high-throughput viral load testing platforms situated in laboratories at selected referral and provincial laboratories [5]. However, HIVDR testing is still limited and is being performed at the National Microbiology Reference laboratory and a few private laboratories.

The WHO HIVDR report of 2021 highlighted significant progress in HIVDR surveillance implementation [6]. However, pretreatment HIVDR to non-nucleoside reverse-transcriptase inhibitors (NNRTI) remained a concern, affecting more than 10% of adults starting therapy and being found to be 3 times more likely in people who had previous exposure to antiretroviral drugs. Notably, half of infants newly diagnosed with HIV exhibited NNRTI resistance before initiating treatment [6].

In Zimbabwe, pretreatment drug resistance to NNRTI was high (15%) whilst pretreatment drug resistance to NRTI was low (4%) [7]. Among adults failing NNRTI-based first-line ART, the levels of resistance to NNRTI and NRTI ranged from 50% to 97% [2,8,9]. The high prevalence of NNRTI resistance is attributed to their low genetic barrier to resistance and their mechanism of action, which targets the reverse transcriptase enzyme that is prone to mutations. Studies have shown that individuals naïve to ART who begin first-line ART also experience HIVDR issues [10,11]. The treatment success for those on the first-line ART regimen is limited by this circumstance. The effectiveness of second-line ART medications would also be impacted by resistance to first-line ART medications [9,12]. This is indicated in recent studies where the effectiveness of second-line regimens is shown to be hampered by a high rate of drug resistance [8,12]. Due to the noted high magnitude of resistance to NRTI and NNRTI, the WHO recommended DTG-based antiretroviral therapy as the preferred first-line regimen for people living with HIV in 2019. The WHO also recommended monitoring HIVDR to DTG in surveys. However, as per the WHO 2024 report, Zimbabwe had not yet conducted a survey to assess HIVDR to DTG [13]. The emergence of HIVDR is a complex process that involves several factors such as adherence to treatment, the potency of the antiretroviral drugs, the genetic diversity of the virus, the duration of treatment, age of patients, marital status, baseline CD4 count, and level of education [14,15]. Identifying these predictors is crucial for understanding the factors that contribute to the development of unsuppressed HIV viral load and drug resistance and to informing the development of more effective treatment strategies [16]. Additionally, it allows for the identification of individuals who are at a higher risk of developing HIVDR. This study aims to identify the predictors of HIVDR among ZIMPHIA 2020 study participants. The findings can help inform healthcare providers and policymakers in developing targeted interventions to prevent drug resistance and improve patient outcomes [3,5].

## 2. Materials and Methods

The methodology of ZIMPHIA 2020 has been described elsewhere [3]. Briefly, ZIMPHIA surveys were nationally representative, cross-sectional population-based surveys of households across Zimbabwe using a stratified multistage probability sampling design. For ZIMPHIA 2020, the first stage selected 356 enumeration areas (EAs) systematically with probability sampling proportional to size, where the size of an EA was defined by the number of households in that EA based on population projections for 2020, derived from the 2012 census. The EAs were stratified by urban–rural status and then geographically within urban–rural status prior to sample selection. During the second stage, a sample of households was randomly selected within each EA, or cluster, using an equal probability method, where the average number of households selected per cluster would be 35. Lastly, in each sample household, all eligible persons who were 15 years or older and were present in the household the night prior to the interview were included in the study. Written informed consent was obtained and an electronic-based individual questionnaire was interviewer administered.

Blood for biomarkers was collected and HIV testing was conducted at households following the Zimbabwe national HIV testing algorithm. Samples that were positive for HIV were shipped to the central laboratory and confirmed using the Genius HIV 1/2 Supplemental Assay (Bio-Rad, Hercules, CA, USA).

To determine the extent of HIV-1 drug resistance mutations among participants in ZIMPHIA 2020, samples from all HIV-positive participants with a viral load ≥ 200 copies/mL were evaluated using a TaqMan^®^ SNP Genotyping Assay (Applied Biosystems), Waltham, MA, USA, to identify mutations within the HIV-1 pol gene region encoding protease, reverse transcriptase, and integrase, which confer resistance and are known to be responsible for resistance to specific ARVs (according to the Stanford University HIV Drug Resistance Database). The testing was performed at National Institute for Communicable Diseases (NICD) in South Africa, with support provided by CDC Atlanta’s International Laboratory Branch, a World Health Organization (WHO)-accredited laboratory for HIVDR testing [3]. ARVs were detected in DBS samples by means of high-resolution liquid chromatography coupled with tandem mass spectrometry. Only four ARVs (efavirenz, nevirapine, atazanavir, and dolutegravir) were detected as markers of commonly prescribed first- and second-line regimens due to the high cost of the test and the relatively long half-life of these ARVs. The test was performed by the Division of Clinical Pharmacology of the Department of Medicine at the University of Cape Town, South Africa, and the detection of ARVs indicated current use of the drug at blood collection time [3].

We conducted secondary data analysis on 673 participants from ZIMPHIA 2020 who were HIV positive and were successfully tested for any form of HIVDR using STATA Version 18, Texas, USA.

Simple proportions were used to describe the baseline demographic characteristics. Pearson chi-square tests were performed to determine the factors associated with any form of HIVDR. Simple logistic regression was used for risk estimation and the odds ratio, and their 95% confidence intervals are presented. Thereafter, adjusted logistic regression was conducted on each variable controlling for age, gender, area of residence, and wealth quintile. Significance level was kept at *p* = 0.05.

## 3. Results

**Figure 1:**
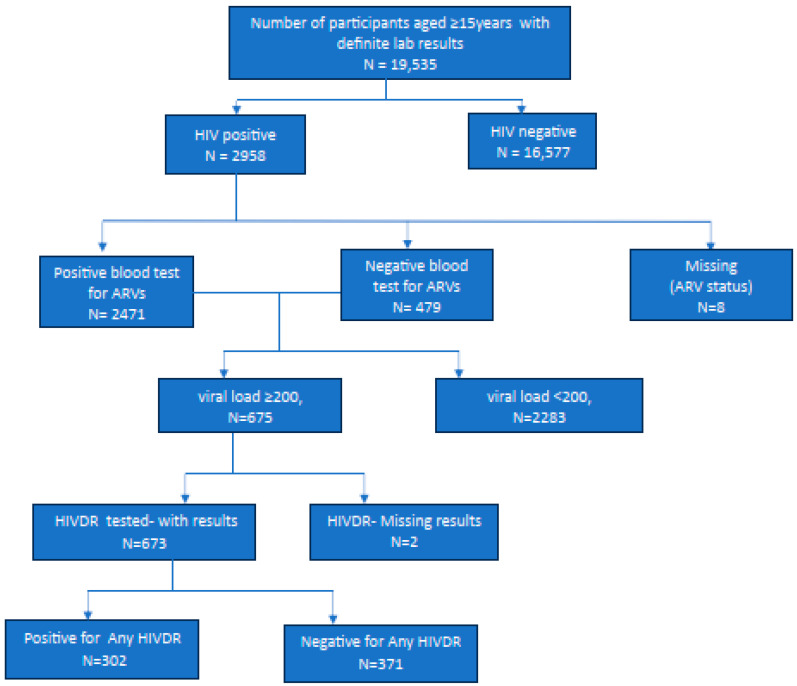
Flow diagram of inclusion of ZIMPHIA 2020 participants tested for HIVDR.

Out of a total of 673 HIV positive participants who were successfully assessed for any form of HIVDR, 302 (44.9%) had some form of drug resistance. Among these, 280 (41.6%) were on ART as confirmed by laboratory tests and 393 were ART naïve, as in Figure 1 below.

Most participants were between the ages of 35–49 years old (n = 273, 40.6%), were female (n = 428, 63.6%), and resided in rural areas (n = 455, 67.6%). The median (interquartile range) age for the participants was 37 (15–73) years. Furthermore, more than half were married (n = 363, 62.6%). The majority (25.7%) of the drug resistance category was NRTI and NNRTI, followed by NNRTI only (17.5%). Further details are presented in Table 1.

The odds of HIVDR were lower in the 50+ age group compared to the 15–24 years age group. Participants who used condoms with a non-marital irregular partner were 55% less likely to have HIVDR compared to those who did not use condoms. This association remained statistically significant for the ART-experienced population. Participants who had undergone an HIV viral load test were about two and half times more likely to have HIVDR compared to those who never had an HIV viral load test. Participants who had switched drug regimens were more likely to have HIVDR compared to those who had not. Participants with a CD4 count of less than 350 were 1.88 times more likely to have HIVDR than those with a CD4 greater than 350. This association remained present after splitting by ART status. Additionally, participants who had the infection for longer or had been on ART for longer were more likely to have HIVDR. This duration factor was not significant among the ART-naïve participants. In the adjusted logistic regression, the notable factors that remained significantly associated with drug resistance were age, number of lifetime sexual partners, condom use at last sexual encounter, duration in years since last HIV positive result, duration in years on ART, ever-switching ARV regimen, and CD4 count of below 350. Chronic conditions such as diabetes, hypertension, and others were also not associated with HIVDR. Refer to Table 2 for more details.

## 4. Discussion

The objectives of this study were to identify the predictors of HIVDR among ZIMPHIA 2020 study participants. A total of 673 samples were sequenced for HIVDR and 302 (44.9%) had at least one major HIVDR mutation detected. Acquired drug resistance was 76.1%, and this is the same as that reported by Chimbetete et al. [8], who in their study found 73% of participants failed second-line ART with at least one major PI mutation. Both studies show that acquired HIV drug resistance is very high in Zimbabwe and there is a need to continue managing and monitoring patients taking ARVs to ensure adherence and improved patient outcomes. The ZIMPHIA 2020 results are lower than what was found by Kouamou et al. (2019) in their study, where 94% of the participants failing first-line treatment had major drug resistance mutations [17]. This suggests that early detection and treatment initiation may be contributing to lower resistance rates.

It was also noted that more females 47.4% had HIVDR mutations than males, 40.4%, although this was not significant (*p* = 0.080) However, these results are consistent with what the WHO found in 2018, where women starting first-line ARV treatment were twice as more likely to have the resistant virus than men [18]. This could be due to factors such as socioeconomic factors, which act as barriers for women to access and adhere to treatment, gender-specific conditions like pregnancy and breastfeeding, which can complicate ART management, challenges in ART adherence due to caregiving responsibilities, stigma and lack of support, and use of ARVs in the prevention of the transmission of HIV from the mother to her child, among other factors [6,19]. However, gender was not a statistically significant predictor of drug resistance (*p* = 0.078) in this study. These results are similar to what was found in other studies, where gender was not significantly associated with HIVDR [7,20]. While our findings suggest that gender may not be a significant predictor of HIVDR at a population level, the observed trend of higher resistance among females warrants further investigation. Factors such as socioeconomic disparities, gender-specific health challenges, and cultural norms could potentially influence treatment adherence and, consequently, HIVDR.

This study also noted that there was no statistically significant differences between most age groups and HIVDR. However, the 50+ age group was 0.57 times less likely to have HIVDR, with a statistical significance of *p* = 0.044. This factor became not significant when the participants were categorized according to ART status. This is consistent with a study conducted by Merik et al. [21], which found that increased ART outcomes increased with age. Growing older could indicate more maturity, stability in one’s lifestyle, and more education relevant to HIVand AIDS; these aspects may probably have an impact on ART therapy compliance. However, these results are different from what Chimbetete et al. [8] found, where age > 24 years was significantly associated with major PI mutations; Ekong et al. [14] also found that older age (31 years+) was significantly associated with HIVDR compared to young age (17 years to 30 years). The discrepancy in age-related HIVDR findings between our study and previous research [5,11] could be due to differences in study populations, geographical locations, or evolving treatment guidelines. It is essential to consider the specific context of each study when interpreting these results.

It was also noted that those who were on ART for a long period were more likely to have HIVDR compared to those who were on ART for a shorter period (odds ratio 1.14). This is consistent with other studies which noted that HIVDR was high in people who were on ART for a longer time, as they were likely to have challenges with ART adherence and prone to drug interruption due to factors such as supply chain challenges, especially in developing countries [14,22,23,24].

Participants who had switched ARVs before had a higher odds ratio (2.53, 95% CI: 1.55–4.14) of having HIVDR than those who had never switched ARV. This is consistent with what was found by Musengimana et al. [25], where patients who had switched ART regimen had a higher odds ratio of HIVDR. It was discovered that there was a strong association between the development of HIVDR mutations and switching ART treatment. This could be the consequence of patients changing their regimens due to ART toxicity or adverse effects. From the time of clinical side effects or toxicity until the time of switching the ART regimen, resistance may occur due to the taking of ARVs in an erratic manner. This study also found that individuals who use condoms with a nonregular partner were 55% less likely to have drug resistance compared to those who do not use condoms. However, after controlling for age, gender, area of residence, and wealth quintile in the adjusted analysis, the effect became insignificant. Our findings highlight the importance of promoting condom use, especially among individuals with drug resistance, and public health interventions should address barriers to condom use. It is also imperative to explore other factors influencing condom use in the context of drug resistance. Although we could not find studies which compared HIVDR and condom use, other studies have found that always using a condom was protective against virological failure, which often led to the development of HIVDR [26].

Participants who previously had a viral load test before showed higher odds of having HIVDR than those who had never tested for viral load. This could be due to the fact that most of them had been on ART treatment before the treat-all policy was introduced by the WHO in 2017. It was also noted that participants who had a CD4 count <350 had a higher risk of having HIVDR than participants who had a CD4 count ≥350. This is consistent with what other studies found: that low CD4 was associated with HIVDR [8,14,27,28].

This underscores the importance of early ART initiation in individuals with low CD4 counts to prevent the emergence of drug-resistant strains. Unlike in the past were someone was initiated on ART based on CD4 count, ART initiation is currently started as soon as someone tests positive.

The analysis did not identify an association between HIVDR and the time traveled to obtain ARV or area of residence, categorized as rural or urban. These findings are divergent from those of Meriki et al. 2014 [21], who found that longer distances from treatment sites was associated with poor outcomes of HIV treatment. The discrepancy can be attributed to initiatives implemented by the Ministry of Health and Child Care in partnership with various local and international partners to establish a person-centered care approach known as a differentiated service delivery (DSD) model to ensure access to ART even in remote areas, including the provision of various support mechanisms. The DSD model streamlines and adapts HIV services throughout the cascade to meet the needs and preferences of diverse populations of HIV-positive people and at-risk individuals while lowering needless strain on the healthcare system. DSD models include mobile outreach, community ART refill groups (CARG), family ART pickups, the House of Smiles (for those of no fixed abode), the O’Malayitsha model for mobile populations, and the fast-track [29,30]. The village health workers, Community Adolescents Treatment Supporters (CATS), key population peer supporters, adult expert recipients of care, and Young Mentor Mothers (YMM) also help with the distribution of refills to HIV-positive people within their communities and groups. These initiatives have significantly reduced the distance and frequency traveled to health facilities to collect medication, saving money and time [29]. This study did not reveal a significant difference in HIVDR between people who had ever attended school and those who had never attended school. This contrasts with findings from a study by Chimbetete et al. [8], which observed a significant association between education levels and HIVDR. The potential reasons could be the various programs carried out by the government, local and international partners with regard to adherence, viral load testing and other programs which aim to reduce the impact of HIV and AIDS and the process addressing risk factors of HIVDR. These programs are tailor-made to address people of all levels of education. The other possible reasons could be due to the difference in the population sequenced in our study compared to the one carried out by Chimbetete et al. [8], where only patients who were failing second-line therapy were sequenced.

With regard to the number of lifetime partners and drug resistance, there was no statistical significance in bivariate analysis nor when participants were categorized according to ART status. However, in adjusted analysis, after controlling for age, gender, area of residence, and wealth quintile, the association became significant. This suggests that having more lifetime sexual partners may be associated with an increased risk of drug resistance and, hence, it is imperative that healthcare providers counsel patients about safer sexual practices.

There was also no significant association between factors such as ever worked, husband has more than one wife, wife has husband with more than one wife, age of sexual debut, mean number of sexual partners in the last 12 months, drinks alcohol, or wealth quintile and any form of HIVDR. A possible explanation could be the impact of various education mechanisms implemented in the ART provision. Although people still engage in risky behavior, it seems they are still taking all the necessary preventive measures to reduce the possibility of developing HIVDR. We also found out that among the ART-naïve participants, 34.45% of the participants who had HIVDR had sex while drunk as compared to 18,0% of the participants who did not have sex while drunk, and the difference was statistically significant. However, sex while drunk was not statistically significantly associated with HIVDR in the bivariate analysis, the adjusted analysis, or among participants already taking ART. The possible reason for ART-naïve participants engaging in risky behavior could be the lack of awareness of their HIV status and hence a continued engaging in risky behaviors. They also do not have the opportunity to participate in educational programs when on ART, and this can contribute to the continuing of engaging in risky behavior. This study also found no significant association between HIVDR and the following conditions: mental health, hypertension, heart disease, kidney disease, lung cancer, and other chronic conditions.

This study contributes to existing data on the factors associated with HIVDR in Zimbabwe. However, the findings should be interpreted with the following limitations in view: testing for HIVDR was limited to the only time the surveillance was carried out. The cause or timing of drug resistance was not considered. Instead of being a randomized clinical trial, this study is a cross-sectional, population-based surveillance project that tracks HIVDR.

## 5. Conclusions

The prevalence of HIVDR mutation was high at 44.9%, and the significant predictors of drug resistance were duration of having a HIV positive result, duration on ART, having ever switched ARVs, previous viral load test, CD4 count, and condom use at last sexual encounter. These parameters were significantly associated with any form of drug resistance mutation. However, factors such as education level and distance traveled to collect ART, among other social parameters, were not significantly associated with any form of HIVDR. This could be attributed to various programs in place to curb HIV as Zimbabwe moves towards epidemic control.

## Figures and Tables

**Table 1:** Baseline demographic characteristics of HIV-infected individuals who were assessed for drug resistance from the ZIMPHIA 2020 survey, N = 673.

Variable	Participants Assessed for Any DR (2020) N = 673 n (%)
Any drug resistance	
No	371 (55.1)
Yes	302 (44.9)
On ART (laboratory tests confirmed)	
Yes	280 (41.6)
No	393 (58.4)
Any drug resistance among participants on ART	
Yes	213 (76.1)
No	67 (23.9)
Any drug resistance among ART naïve participants	
Yes	89 (22.6)
No	304 (77.4)
Drug resistance category	
NRTI Only	2 (0.3)
NNRTI Only	118 (17.5)
PI Only	1 (0.2)
INSTI Only	1 (0.2)
NRTI and NNRTI	173 (25.7)
NRTI and NNRTI and PI	6 (0.9)
Other	1 (0.2)
None	371 (55.1)
Age in years	
15–24	104 (15.5)
25–34	186 (27.6)
35–49	273 (40.6)
50+	110 (16.3)
Gender	
Male	245 (36.4)
Female	428 (63.6)
Area	
Urban	218 (32.4)
Rural	455 (67.6)
Ever attended school	
Yes	653 (97.2)
No	19 (2.8)
Ever worked	
Yes	314 (46.7)
No	359 (53.3)
Current marital status	
Married	363 (62.6)
Living together	23 (4.0)
Widowed	88 (15.2)
Divorced	51 (8.8)
Separated	55 (9.5)
Wealth quintile	
Poorest	166 (24.7)
Second	136 (20.2)
Middle	116 (17.2)
Fourth	121 (18.0)
Richest	134 (19.9)

**Table 2:** Predictors of drug resistance among HIV-positive participants from ZIMPHIA 2020 survey, N = 673.

	All (N = 673)						Among Participants on ART (N = 280)			Among ART-Naïve Participants (N = 393)		
Variable	Has Any Drug Resistance N = 302	No Drug Resistance N = 371	Odds Ratio (OR) (95% Confidence Interval)	*p* Value	* Adjusted Odds Ratio (aOR) (95% Confidence Interval)	*p* Value	Has Any Drug Resistance N = 213	No Drug Resistance N = 67	*p* Value	Has Any Drug Resistance N = 89	No Drug Resistance N = 304	*p* Value
Age in years												
15–24	54 (51.9)	50 (48.1)	1		1		34 (85.0)	6 (15.0)		20 (31.3)	44 (68.8)	
25–34	78 (41.9)	108 (58.1)	0.67 (0.41–1.08)	0.102	0.63 (0.38–1.02)	0.062	49 (71.0)	20 (29.0)		29 (24.8)	88 (75.2)	
35–49	128 (46.9)	145 (53.1)	0.82 (0.52–1.28)	0.382	0.79 (0.50–1.25)	0.317	96 (77.4)	28 (22.6)		32 (21.5)	117 (78.5)	
50+	42 (38.2)	68 (61.8)	0.57 (0.33–0.99)	0.044	0.56 (0.32–0.98)	0.040	34 (72.3)	13 (27.7)	0.361	8 (12.7)	55 (87.3)	0.083
Gender												
Male	99 (40.4)	146 (59.6)	1		1		70 (75.3)	23 (24.7)		29 (19.1)	123 (80.9)	
Female	203 (47.4)	225 (52.6)	1.33 (0.97–1.83)	0.078	1.34 (0.97–1.85)	0.080	143 (76.5)	44 (23.5)	0.824	60 (24.9)	181 (75.1)	0.180
Area												
Urban	94 (43.1)	124 (56.9)	1		1		65 (74.7)	22 (25.3)		29 (22.1)	102 (77.9)	
Rural	208 (45.7)	247 (54.3)	1.11 (0.80–1.54)	0.527	1.32 (0.72–2.42)	0.373	148 (76.7)	45 (23.3)	0.720	60 (22.9)	202 (77.1)	0.865
Ever attended school												
Yes	291 (44.6)	362 (55.4)	1		1		204 (75.6)	66 (24.4)		87 (22.7)	296 (77.3)	
No	11 (57.9)	8 (42.1)	1.71 (0.68–4.31)	0.255	1.86 (0.72–4.79)	0.197	9 (100.0)	0 (0.0)	0.090	2 (20.0)	8 (80)	0.839
Ever worked												
Yes	139 (44.3)	175 (55.7)	1		1		102 (75.0)	34 (25.0)		37 (20.8)	141 (79.2)	
No	163 (45.4)	196 (54.6)	1.05 (0.77–1.42)	0.767	1.11 (0.81–1.52)	0.528	111 (77.1)	33 (22.9)	0.683	52 (24.2)	163 (75.8)	0.423
Age of sexual debut in years, Median (IQR)	18 (16–20)	18 (16–20)	0.98 (0.93–1.02)	0.335	0.99 (0.94–1.04)	0.766	18 (16–21)	18 (16–21)	0.826	18 (16–21)	18 (17–21)	0.062
Number of lifetime sexual partners, Median (IQR)	3 (2–5)	3 (2–5)	1.02 (0.99–1.04)	0.177	1.03 (1.01–1.06)	0.017	3 (2–5)	2 (2–5)	0.882	3 (2–6)	3 (2–5)	0.063
Mean number of sexual partners in the last 12 months, Median (IQR)	1 (0–1)	1 (0–1)	0.99 (0.83–1.17)	0.896	0.99 (0.82–1.18)	0.880	1 (0–1)	1 (0–1)	0.664	1 (1–1)	1 (1–1)	0.029
Condom use at last sexual encounter												
Yes	88 (53.0)	78 (47.0)	1		1		76 (78.3)	21 (21.7)		12 (17.4)	57 (82.6)	
No	103 (36.3)	181 (63.7)	0.50 (0.34–0.74)	<0.001	0.49 (0.33–0.73)	0.001	57 (72.2)	22 (27.8)	0.341	46 (22.4)	159 (77.6)	0.375
Condom use with nonmarital nonregular partner												
No	45 (51.7)	42 (48.3)	1		1		32 (86.5)	5 (13.5)		13 (26.0)	37 (74.0)	
Yes	23 (32.4)	48 (67.6)	0.45 (0.23–0.86)	0.015	0.53 (0.26–1.06)	0.073	11 (57.9)	8 (42.1)	0.016	12 (23.1)	40 (76.9)	0.732
Drinks alcohol												
No	230 (46.6)	264 (53.4)			1		168 (76.4)	52 (23.6)		62 (22.6)	212 (77.4)	
Yes	71 (40.1)	106 (59.9)	0.77 (0.54–1.09)	0.140	0.89 (0.60–1.31)	0.547	44 (74.6)	15 (25.4)	0.775	27 (22.9)	91 (77.1)	0.956
Sex while drunk												
No	137 (40.2)	204 (59.8)	1		1		100 (74.1)	35 (25.9)		37 (18.0)	169 (82.0)	
Yes	52 (51.0)	50 (49.0)	1.55 (0.99–2.42)	0.054	1.52 (0.96–2.39)	0.074	30 (79.0)	8 (21.1)	0.539	22 (34.4)	42 (65.6)	0.006
Duration in years since first HIV positive result, Median (IQR)	8 (4–11)	5 (2–8)	1.15 (1.08–1.23)	<0.001	1.17 (1.09–1.25)	<0.001	8 (4–11)	2 (5–8)	<0.001	3 (1–9)	3 (1–9)	0.968
Ever had a viral load test												
No	81 (58.3)	58 (41.7)	1		1		58 (70.7)	24 (29.3)				
Yes	138 (78.0)	39 (22.0)	2.53 (1.55–4.14)	<0.001	2.54 (1.54–4.17)	<0.001	138 (80.2)	34 (19.8)	0.092			
Viral load, Count Median (IQR)	19,348 (3214–64,722)	25,788 (7387–78,712)	0.99 (0.99–1.00)	0.305	0.99 (0.99–1.00)	0.301	12980 (2152–46,907)	5379 (622–26,871)	0.046	41,168 (14,295–100,591)	31,193 (11,654–87,237)	0.666
CD4 Count												
≥350	100 (35.8)	179 (64.2)	1		1		70 (67.3)	34 (32.7)		30 (17.1)	145 (82.9)	
<350	202 (51.3)	192 (48.7)	1.88 (1.38–2.58)	<0.001	2.04 (1.48–2.83)	<0.001	143 (81.3)	33 (18.8)	0.008	59 (27.1)	159 (72.9)	0.020
Any TB symptom during last clinic visit												
No	118 (65.6)	62 (34.4)	1		1		105 (76.1)	33 (23.9)		13 (31.0)	29 (69.0)	
Yes	106 (72.6)	40 (27.4)	1.39 (0.86–2.24)	0.173	1.37 (0.84–2.23)	0.206	96 (78.1)	27 (22.0)	0.707	10 (43.5)	13 (56.5)	0.313
Travel time in hours to pick ARV, Median IQR	2 (1–3_	2 (1–3)	1.17 (0.90–1.52)	0.246	1.14 (0.85–1.52)	0.381	2 (1–3)	2 (1–3)	0.196	2 (1–4)	3 (2–3)	0.661
Having little interest in doing things in last two weeks												
No	180 (42.4)	245 (57.7)	1		1		120 (74.1)	42 (25.9)		60 (22.8)	203 (77.2)	
Yes	122 (49.6)	124 (50.4)	1.34 (0.98–1.84)	0.070	1.32 (0.96–1.82)	0.086	93 (78.8)	25 (21.2)	0.359	29 (22.7)	99 (77.3)	0.972
Ever had diabetes												
No	296 (45.1)	361 (55.0)	1		1		208 (75.6)	67 (24.4)		88 (23.0)	294 (77.0)	
Yes	5 (38.5)	8 (61.5)	0.76 (0.25–2.35)	0.637	0.72 (0.23–2.26)	0.571	4 (100.0)	0 (0.0)	0.257	1 (11.1)	8 (88.9)	0.399
Ever had hypertension												
No	278 (46.1)	325 (53.9)	1		1		194 (75.8)	62 (24.2)		84 (24.2)	263 (75.8)	
Yes	23 (34.3)	44 (65.7)	0.61 (0.36–1.04)	0.068	0.62 (0.36–1.08)	0.093	18 (78.3)	5 (21.7)	0.790	5 (11.4)	39 (88.6)	0.056
Ever had heart disease												
No	299 (45.0)	366 (55.0)	1		1		211 (76.2)	66 (23.8)		88 (22.7)	300 (77.3)	
Yes	2 (40.0)	3 (60.0)	0.82 (0.14–4.92)	0.824	0.84 (0.14–5.11)	0.846	1 (50.0)	1 (50.0)	0.388	1 (33.3)	2 (66.7)	0.661
Ever had kidney disease												
No	299 (45.0)	366 (55.0)	1		1		210 (75.8)	67 (24.2)		89 (22.9)	299 (77.1)	
Yes	2 (40.0)	3 (60.0)	0.82 (0.14–4.92)	0.824	0.81 (0.13–5.01)	0.823	2 (100.0)	0 (0.0)	0.425	0 (0.0)	3 (100.0)	0.345
Ever had cancer												
No	299 (44.9)	367 (55.1)	1		1		211 (75.9)	67 (24.1)		88 (22.7)	300 (77.3)	
Yes	2 (50.0)	2 (50.0)	1.23 (0.17–8.77)	0.838	1.41 (0.19–10.22)	0.735	1 (100.0)	0 (0.0)	0.573	1 (33.3)	2 (66.7)	0.661
Lung disease												
No	299 (45.0)	365 (55.0)	1		1		210 (76.4)	65 (23.6)		89 (22.9)	300 (77.1)	
Yes	2 (33.3)	4 (66.7)	0.61 (0.11–3.36)	0.570	0.58 (0.10–3.35)	0.546	2 (50.0)	2 (50.0)	0.220	0 (0.0)	2 (100.0)	0.441
Mental health problem												
No	300 (44.8)	369 (55.2)	1		1		211 (75.9)	67 (24.1)		89 (22.8)	302 (77.2)	
Yes	1 (100.0)	0 (0.0)	Omitted	-	Omitted	-	1 (100.0)	0 (0.0)	0.573	-	-	-
Other chronic conditions												
No	299 (44.8)	368 (55.2)	1		1		211 (75.9)	67 (24.1)		88 (22.6)	301 (77.4)	
Yes	2 (66.7)	1 (33.3)	2.46 (0.22–27.28)	0.463	2.07 (0.18–23.44)	0.557	1 (100.0)	0 (0.0)	0.573	1 (50.0)	1 (50.0)	0.357
Wealth quintile												
Poorest	78 (47.0)	88 (53.0)	1		1		62 (78.5)	17 (21.5)		16 (18.4)	71 (81.6)	
Second	64 (47.1)	72 (52.9)	1.00 (0.64–1.58)	0.990	1.03 (0.65–1.62)	0.906	45 (80.4)	11 (19.6)		19 (23.8)	61 (76.3)	
Middle	46 (39.7)	70 (60.3)	0.74 (0.46–1.20)	0.223	0.77 (0.47–1.26)	0.297	28 (68.3)	13 (31.7)		18 (24.0)	57 (76.0)	
Fourth	56 (46.3)	65 (53.7)	0.97 (0.61–1.55)	0.906	1.19 (0.63–2.27)	0.587	40 (74.1)	14 (25.9)		16 (23.9)	51 (76.1)	
Richest	58 (43.3)	76 (56.7)	0.86 (0.54–1.36)	0.522	1.00 (0.48–2.07)	0.996	38 (76.0)	12 (24.0)	0.681	20 (23.8)	64 (76.2)	0.885
**Among Participants on ART**												
On ART (laboratory tests confirmed)												
Yes	213 (76.1)	67 (23.9)	1		1							
No	89 (22.7)	304 (77.4)	0.09 (0.06–0.13)	<0.001	0.09 (0.06–0.13)	<0.001	-	-	-	-	-	-
Duration in years on ART, Median (IQR)	7.5 (4–10)	5 (2–7)	1.14 (1.06–1.22)	<0.001	1.14 (1.06–1.23)	<0.001	-	-	-	-	-	-
Date of ART initiation												
After 2014	90 (64.3)	50 (35.7)	1		1							
Before 2014	116 (84.1)	22 (15.9)	2.93 (1.65–5.19)	<0.001	3.08 (1.72–5.49)	0.020	-	-	-	-	-	-
Ever switched ARVs												
No	132 (73.3)	48 (26.7)	1		1							
Yes	65 (86.7)	10 (13.3)	2.36 (1.12–4.97)	0.023	2.47 (1.15–5.29)	0.020	-	-	-	-	-	-

* In the adjusted logistic regression, the variables controlled for were age, gender, area of residence and wealth quintile.

## Data Availability

All data are available on reasonable request from ICAP at Columbia university https://phia-data.icap.columbia.edu (accessed on 22 October 2024).
